# Epothilone B Speeds Corneal Nerve Regrowth and Functional Recovery through Microtubule Stabilization and Increased Nerve Beading

**DOI:** 10.1038/s41598-018-20734-1

**Published:** 2018-02-08

**Authors:** Hanqing Wang, Chengju Xiao, Dong Dong, Cuipei Lin, Yunxia Xue, Jun Liu, Mingjuan Wu, Jingxin He, Ting Fu, Hongwei Pan, Xinwei Jiao, Dingli Lu, Zhijie Li

**Affiliations:** 1 International Ocular Surface Research Center, Institute of Ophthalmology, Key Laboratory for Regenerative Medicine of the Ministry of Education, Jinan University, Guangzhou, China; 2 Henan Key Laboratory of Ophthalmology and Visual Science, People’s Hospital of Zhengzhou University, Henan University School of Medicine, Henan Provincial People’s Hospital, Zhengzhou, China; 3 Department of Immunology and Microbiology, Jinan University Medical School, Guangzhou, China; 4 Section of Leukocyte Biology, Department of Pediatrics, Children’s Nutrition Research Center, Baylor College of Medicine, Houston, Texas USA

## Abstract

The successful restoration of corneal innervation and function after a corneal injury is a clinically challenging issue. Structural and functional recovery after a nerve injury involves a complex series of steps in which microtubules play a key role. The aim of the current study was to investigate the effects of epothilone B (EpoB), a microtubule-stabilizing agent, on corneal innervation and the functional recovery of the corneal nerve in mice after corneal epithelial abrasion. The pretreatment of mice with EpoB has a remarkable effect on the stabilization of beta-III tubulin, as demonstrated by substantial increases in the visualization of beta-III tubulin, nerve beading, corneal reinnervation, and reaction to stimuli. Furthermore, a pharmacokinetic analysis showed that EpoB remains at a high concentration in the cornea and the trigeminal ganglion for at least 6 days after administration. In addition, the administration of EpoB at 24 hours after corneal abrasion has a marked therapeutic effect on nerve regrowth and functional recovery. In conclusion, EpoB treatment may have therapeutic utility for improving corneal reinnervation and restoring sensitivity following corneal injury.

## Introduction

Located in the anterior segment of the eye, the cornea is the most important ocular medium in the visual system. The diopter of the cornea takes up one third of the whole dioptric system, and any slight change in its structure can cause a significant decrease, or even loss, of visual acuity. In fact, 1.57 million people suffer from corneal blindness worldwide^1^.

As some of the most densely innervated tissues in the body, the corneal nerves are derived from the trigeminal ganglion, which not only perceives stimulation from the external environment^2^ but also provides nutrition to the cornea by producing neural peptides. In addition, recent studies have indicated that the presence of some corneal nerve tissue is important for maintaining the wetness and basal tearing of the ocular surface, as controlled by cold thermoreceptors^3^. However, the corneal nerves are routinely damaged through events that include surgical procedures^4^, corneal infections, the long-term usage of eye medications^5,6^, and systemic diseases such as diabetes^7,8^.

Clinical studies have shown that corneal nerves can regenerate over a period of several years after surgical transection; however, the nerve density never returns to presurgical values^9^. For example, after laser-assisted *in situ* keratomileusis (LASIK) surgery, the subbasal nerve density was found to decrease by 82% within 5 days, and at 2 years postsurgery, the nerve density was still only 64% of its preoperative value^10^. Moreover, subbasal nerve density is not restored to normal levels even 40 years after penetrating keratoplasty^11^, and the median subbasal nerve density in clear grafts is also significantly lower than that of normal corneas^12^.

Despite the fact that there is an urgent clinical need for the promotion of corneal nerve regeneration in neurotrophic corneas, few specific therapeutic interventions are currently available. Some substances such as nerve growth factor^13,14^, neuroprotectin D1^15^, leukemia inhibitory factor^16^, IL-17^17^, and VEGF^17^ are known to promote corneal nerve regeneration through various mechanisms. However, there are currently no drugs available on the market that provide satisfactory corneal nerve restoration. Therefore, the search for new measures to promote nerve repair is imperative.

Microtubules (MTs) are the main cytoskeletal components that support motor-driven cargo transport, neuronal polarity, axon differentiation, and growth in neuronal processes^18,19^. When an axon is injured, MTs are extensively remodeled, leading to the formation and extension of growth cones^20^. In contrast, the disorganization of MTs contributes to dystrophic end bulb formation after injury to the central nervous system (CNS), including the brain and spinal cord^21^. Thus, pharmacologically stabilizing MT dynamics can promote axon regrowth *in vitro* and *in vivo*^22–24^. Structurally, MTs are hollow cylinders composed of alpha- and beta-tubulin heterodimers that join end-to-end to form protofilaments. Beta-III tubulin exerts GTPase activity to hydrolyze guanosine triphosphate (GTP) into guanosine diphosphate (GDP), effectively governing MT formation^25^. Several isoforms of beta-tubulin are expressed in a tissue-specific manner. Beta-II and beta-IV tubulin are ubiquitously expressed, while beta-III tubulin is regarded as a neuron-specific marker. In fact, the expression of beta-III tubulin has been suggested as one of the earliest markers that signals neuronal commitment in the primitive neuroepithelium^26^. The integrity of beta-tubulin is necessary for the formation of the correct number of neurites by a neuron *in vivo* and for an axon to be capable of regeneration^27^. However, the therapeutic effect of MT stabilizers on corneal nerve regeneration and functional recovery has not previously been assessed.

Epothilones were originally identified as secondary metabolites produced by the soil-dwelling mycobacterium *Sorangium cellulosum*^28^. Structurally, their central body is composed of a 16-member macrolide with a thiazole ring ligand as the side chain^29^. Epothilones can promote tubulin polymerization, prevent depolymerization, disturb the growth of tumor cells, and induce cell apoptosis^30^. Furthermore, clinical studies have shown that epothilone B (EpoB) has a therapeutic effect on ovarian cancer, non-small-cell lung cancer, and breast cancer^31^. In 2015, Jörg Ruschel found that EpoB has a powerful effect on regrowth and functional recovery following rodent spinal cord injury^24^. This study suggested that after injury, lesion scars and poor axon growth prevent axon regeneration, and EpoB propels axon growth throughout the scarring area by inducing concerted MT polymerization into the axon tip. Additionally, EpoB reinitiates neuronal polarization by inducing facilitated MT polymerization into the axon tip, which pushes the axonal extension through an inhibitory environment^24^. Recent *in vitro* and *in vivo* observations have shown that the beneficial and detrimental effects of MT stabilization by EpoB depend not only on the drug concentration, but also the type and age of the neurons^32^. Although EpoB can promote axon regeneration and improve motor function after a spinal cord injury, it is unclear whether EpoB accelerates the regeneration of the structure and function of corneal nerves.

In investigating this question, we found that the systemic administration of EpoB can prevent corneal nerve fiber degeneration, stabilize corneal nerves, promote axon regrowth, and increase nerve beading following an injury. In addition, pharmacokinetic (PK) studies demonstrated that after systemic administration, high concentrations of EpoB remained in the cornea and trigeminal ganglion at least for 6 days. Together, these data reveal a promising beneficial effect of the MT stabilizer EpoB on injured corneal nerves.

## Results

### The cornea and trigeminal ganglion sustain high concentrations of EpoB after systemic administration

PK studies can be used to accurately determine the distribution of drugs in various organs and provide evidence for drug availability. To assess the distribution of EpoB in the major organs (heart, liver, spleen, lungs, and kidneys), the CNS (brain and spinal cord), the plasma, and the cornea and trigeminal ganglion, we conducted PK analysis by mass spectrometry following the administration of 1 mg/kg EpoB through i.p. injection.

**Figure 1:**
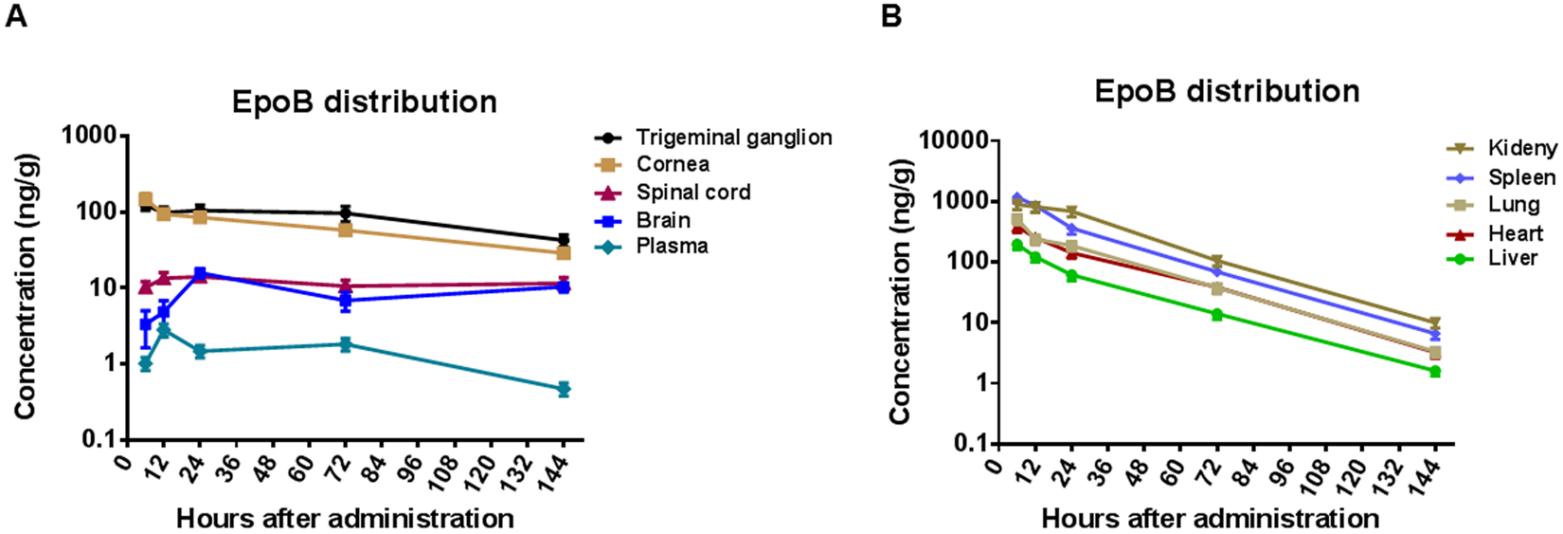
Detailed results of pharmacokinetic (PK) studies of EpoB. **(A)** Concentrations observed in the trigeminal ganglion, cornea, spinal cord, brain, and plasma at various time points after intraperitoneal (i.p.) injection of Epo solution (1 mg/kg). **(B)** PK parameters of EpoB in mouse kidneys, spleen, lungs, heart, and liver after i.p. injection of EpoB solution (1 mg/kg). The results are presented as the mean ± SD. *P < 0.05, **P < 0.01; *n* = 5 mice at each time point (each mouse has two corneas and two trigeminal ganglia).

Our results showed that the spinal cord and brain maintain about 10 ng of EpoB per gram of tissue for more than 144 h, which is consistent with previous studies showing prolonged retention of EpoB in the CNS^24^. In the CNS, the concentration of EpoB was approximately 10 times that seen in the plasma. The peak concentration in the spinal cord (C_max_) was 14.07 ± 2.76 ng/g, and at 144 h (6 d), EpoB remained at 11.54 ± 2.18 ng/g. Similarly, the peak concentration in the brain (C_max_) was 11.77 ± 2.27 ng/g, and the concentration at 144 h was 7.89 ± 1.55 ng/g. Elimination was extremely rare in the brain and spinal cord (Fig. 1A, Table 1). Moreover, in the plasma, the t_max_ of EpoB was 12 h, and the peak concentration (C_max_) was 2.82 ± 0.54 ng/ml. The concentration of EpoB in the plasma was 0.47 ± 0.09 ng/ml at 144 h (6 d; Fig. 1A, Table 1). EpoB showed similar PKs in other tissues (spleen, heart, lungs, liver, and kidneys), with a rapid spread followed by a decline beginning at a similar time point with a mean half-life (t_1/2_) of 21.63 ± 1.16 h (Fig. 1B, Table 1). To determine the distribution of EpoB in the cornea after systemic administration, we removed the corneas with a complete limbus and homogenized the pooled corneas with a homogenizer. EpoB distribution in the cornea was similar to that seen in the brain and spinal cord, with peak concentrations of 148.65 ± 19.68 ng/g at 6 h, followed by a slow elimination (Fig. 1A, Table 1). In the cornea, almost all sensory nerve fibers derive from the ophthalmic division of the trigeminal ganglion. Thus, after i.p. administration, the concentration of EpoB was also measured in the trigeminal ganglion, where it peaked at 122.96 ng/g at 6 h, followed by a slow elimination (Fig. 1A, Table 1). Previously, 0.1 ng/g was shown to be an effective concentration of EpoB in both rats and mice^33^. Thus, the data in this study indicate that EpoB is maintained at an effective drug concentration in the cornea and the trigeminal ganglion for up to 6 days following systemic administration and that the appropriate dosage interval is 6 days or more.

**Table 1:** Pharmacokinetic parameters of EpoB in normal mice after intraperitoneal injection of EpoB solution (1 mg/kg).

Organ	Parameters	t_max_	c_max_	t_1/2_	AUC_0-t_	K_P_
	Units	h	ng/g (ml)	h	ng/g (ml)/h	
Cornea		6	148.65 ± 19.86	66.71	8827.77	43.72
Trigeminal ganglion		6	122.96 ± 18.61	46.98	12114.19	60.00
Plasma		12	2.82 ± 0.54	60.17	201.90	1.00
Spinal cord		24	14.07 ± 2.76	485.35	1657.66	8.21
Brain		24	11.77 ± 2.27	447.74	1325.48	6.57
Spleen		6	1174.87 ± 226.75	20.84	29958.27	148.38
Heart		6	384.98 ± 76.23	21.42	11291.44	55.93
Lung		6	515.53 ± 102.07	22.79	13381.47	66.28
Liver		6	196.86 ± 36.42	22.85	5020.40	24.87
Kidney		6	891.51 ± 162.26	20.25	39923.10	197.73

Kp = AUC0-t_organ_/AUC0-t_plasma_; ng/g for tissues, ng/ml for plasma.

### Systemic administration of EpoB does not influence the gross structure or sensitivity of the cornea, nor does it affect reepithelialization or neutrophil flux to the wound after abrasion

**Figure 2:**
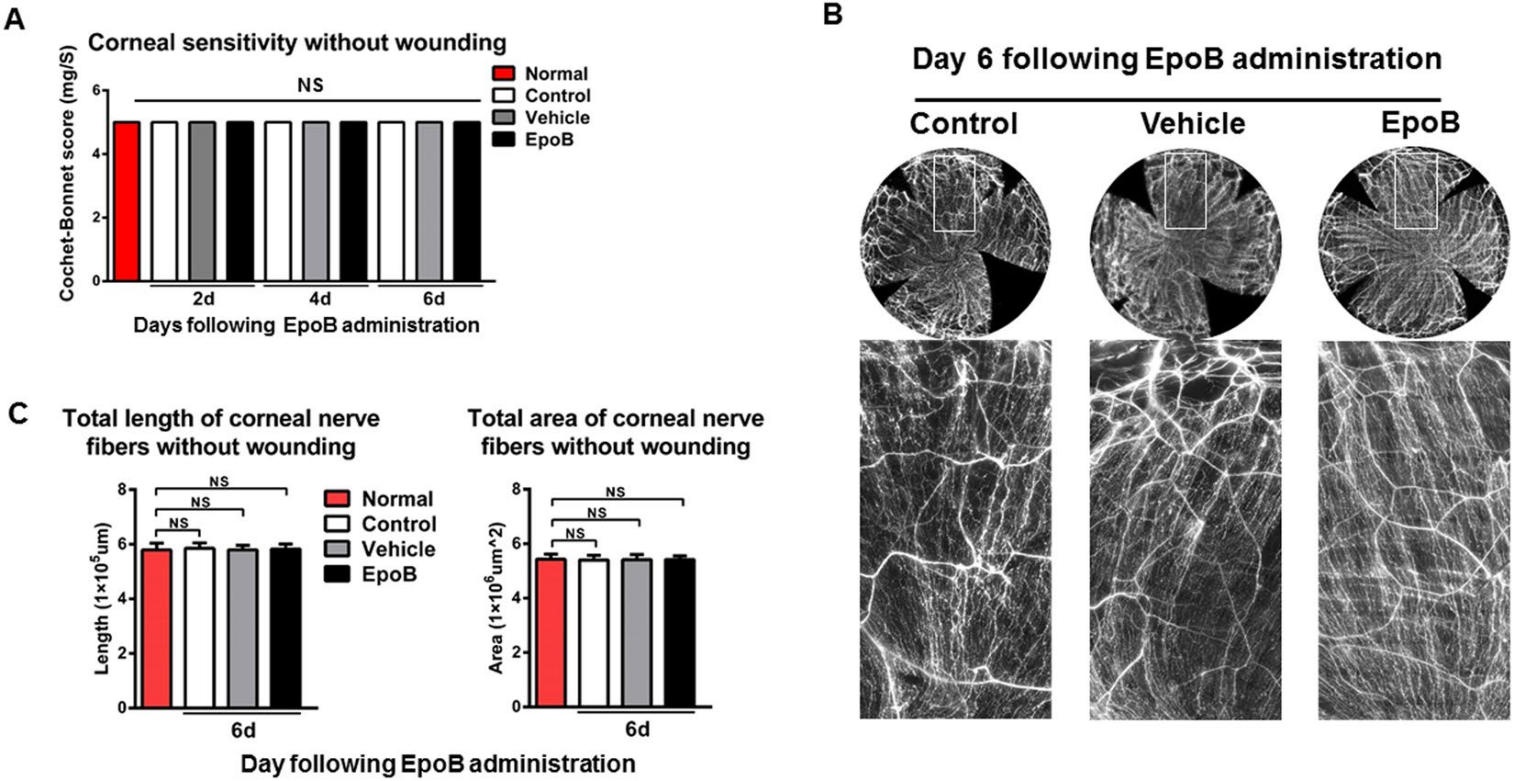
Systemic administration of EpoB does not influence the sensitivity or subbasal nerve density under steady conditions. (**A**) At days 2, 4, and 6 after administration of i.p. EpoB, the corneal sensitivity levels were measured with a Cochet-Bonnet esthesiometer and compared amongst the EpoB, vehicle, and normal groups. (**B**) Photos of complete corneal nerves stained for β-III tubulin were acquired using a DeltaVision Elite high-resolution microscope at day 6 post-i.p. injection of EpoB. **(C)** Changes in the total length and total area of nerve fibers of the central test region (Fig. 10B) in the EpoB, control, and vehicle groups after the administration of i.p. EpoB. The results are presented as the mean ± SD. A factorial design ANOVA was performed to analyze the overall differences between the two groups, and Student’s *t*-test was used to compare the differences between the groups by time point. *P < 0.05, **P < 0.01; *n* = 6 corneas/group. N.S. = not significant by Student’s *t*-test.

EpoB was approved for clinical use as an anticancer drug by the FDA and is believed to exert its anticancer effects mainly through the induction of MT polymerization, the stabilization of MTs against depolymerization, and cytotoxicity in cells overexpressing P-glycoprotein. To find out whether the systemic administration of EpoB causes adverse side effects in the undamaged cornea, mice were given i.p. injections of 1 mg of EpoB per kilogram of body weight, and then *in vivo* corneal sensitivity was determined at days 2, 4 and 6 after injection. The nerve density in fixed tissues in the central test area (2000 µm × 2000 µm) was determined at day 6 after i.p. injection. The results showed that compared with normal control animals, corneal sensitivity (5 ± 0 mg/S, N.S.; Fig. 2A) and nerve density (including nerve length and nerve area) (Fig. 2B,C, and D) did not change.

**Figure 3:**
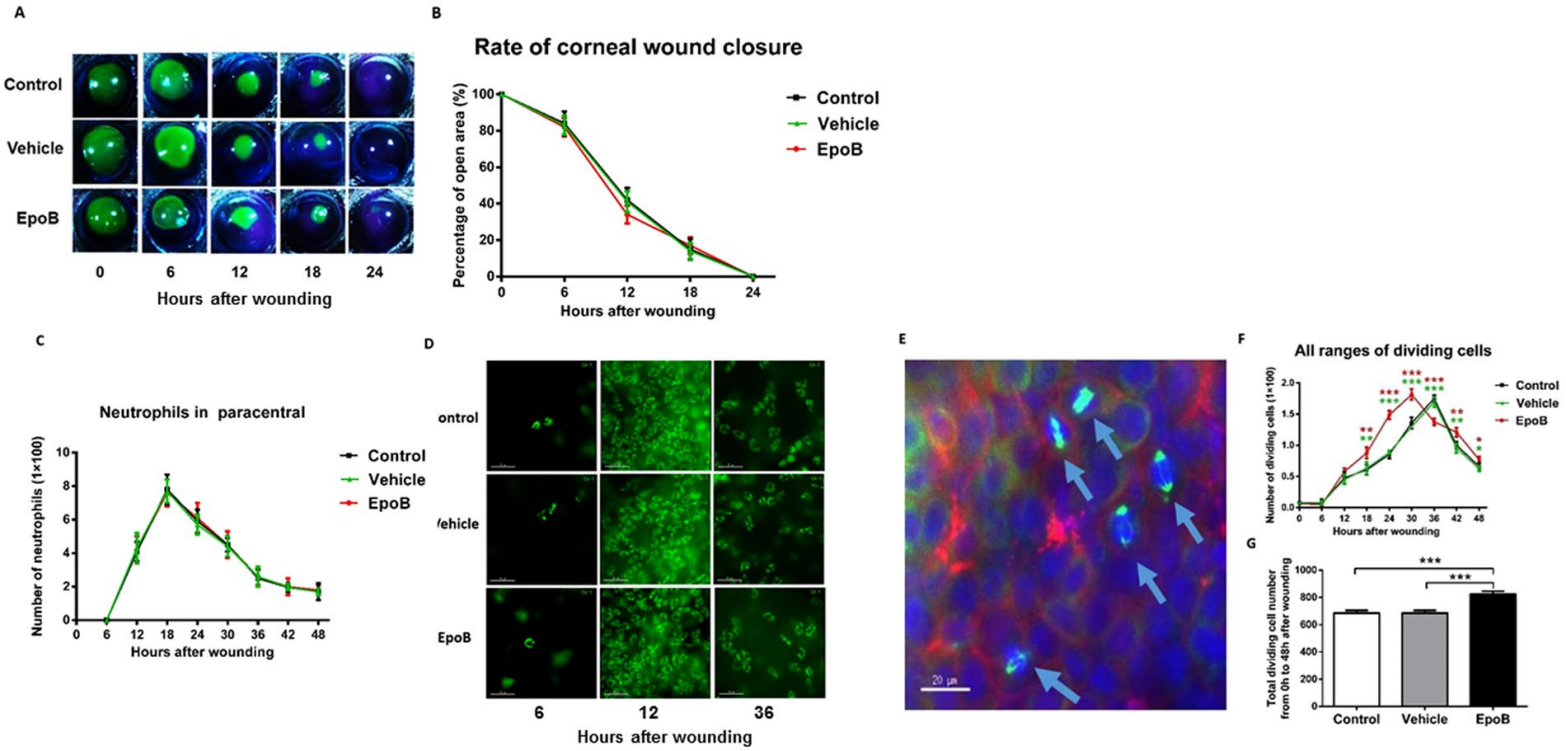
EpoB i.p. injection before injury does not influence the corneal wound healing process. **(A)** The corneal epithelial wounds were stained with sodium fluorescein at the specified time points. **(B)** The percentage of the wound open at each time point was calculated as shown in the line chart (*n* = 6 mice at each time point). **(C)** The number of neutrophils in Zone 4 (Fig. 11) was counted at the specified time point after wounding in the EpoB, vehicle, and normal control groups. **(D)** Corneas were stained with anti-Ly6g-1-FITC (original magnification: 400X). No significant differences were found compared to the vehicle and normal groups. The results are presented as the mean ± SD. **(E)** Corneas were triple-stained with FITC-tagged anti-α-tubulin (green), rhodamine-phalloidin (red), and DAPI (blue; original magnification: 400X). The arrows in Fig. 5E indicate dividing cells with their mitotic spindles stained with FITC-conjugated anti-α-tubulin (green). (**F**) The number of dividing cells was counted at the specified time point after wounding in the EpoB, vehicle, and normal control groups. **(G)** The total number of dividing cells was counted from 0 h to 48 h after wounding in the EpoB, vehicle, and normal control groups. No significant differences were found compared to the vehicle and normal groups. The results are presented as the mean ± SD. A factorial design ANOVA was performed to analyze the differences between the two groups, and Student’s *t*-test was used to compare the replicates by time point. *P < 0.05, **P < 0.01, ***P < 0.001; *n* = 6 corneas/group.

Corneal wound healing is a complex process that mainly involves three continuous but overlapping stages: the reepithelialization, proliferative, and inflammatory stages^34^. To determine whether the systemic administration of EpoB influences the healing process of corneal epithelial abrasions, we first observed the effects of EpoB on three processes of corneal wound repair—reepithelialization, epithelial proliferation, and neutrophil flux to the wounded cornea—using our standard corneal abrasion model. We found that pretreatment with i.p. administration of EpoB at a dose of 1 mg/kg did not influence the reepithelialization (Fig. 3A and B) or neutrophil flux to the wounded cornea (Fig. 3C and D). However, it should be noted that the peak number of dividing cells in the systemic administration group was shifted six hours earlier compared to the normal control group and vehicle group (Fig. 3E and F). The number of dividing cells was significantly greater than in the control group at 18 h (*P* < 0.01), 24 h (*P* < 0.001), 30 h (*P* < 0.001), 42 h (*P* < 0.01), and 48 h (*P* < 0.05). In contrast, the number of dividing cells in the EpoB group was less than that in the control group only at 36 h postwounding (*P* < 0.001; Fig. 3F). The total number of dividing cells at nine time points from 0 to 48 h in the EpoB group was significantly greater than that in the control group (*P* < 0.001; Fig. 3G). Together, these data indicate that the dosage used caused no obvious side effects in steady or wounded corneas. However, EpoB shifted the cell division peak earlier and increased the number of divisions after abrasion.

### EpoB protects axons from injury-induced degeneration at the early stage of injury

**Figure 4:**
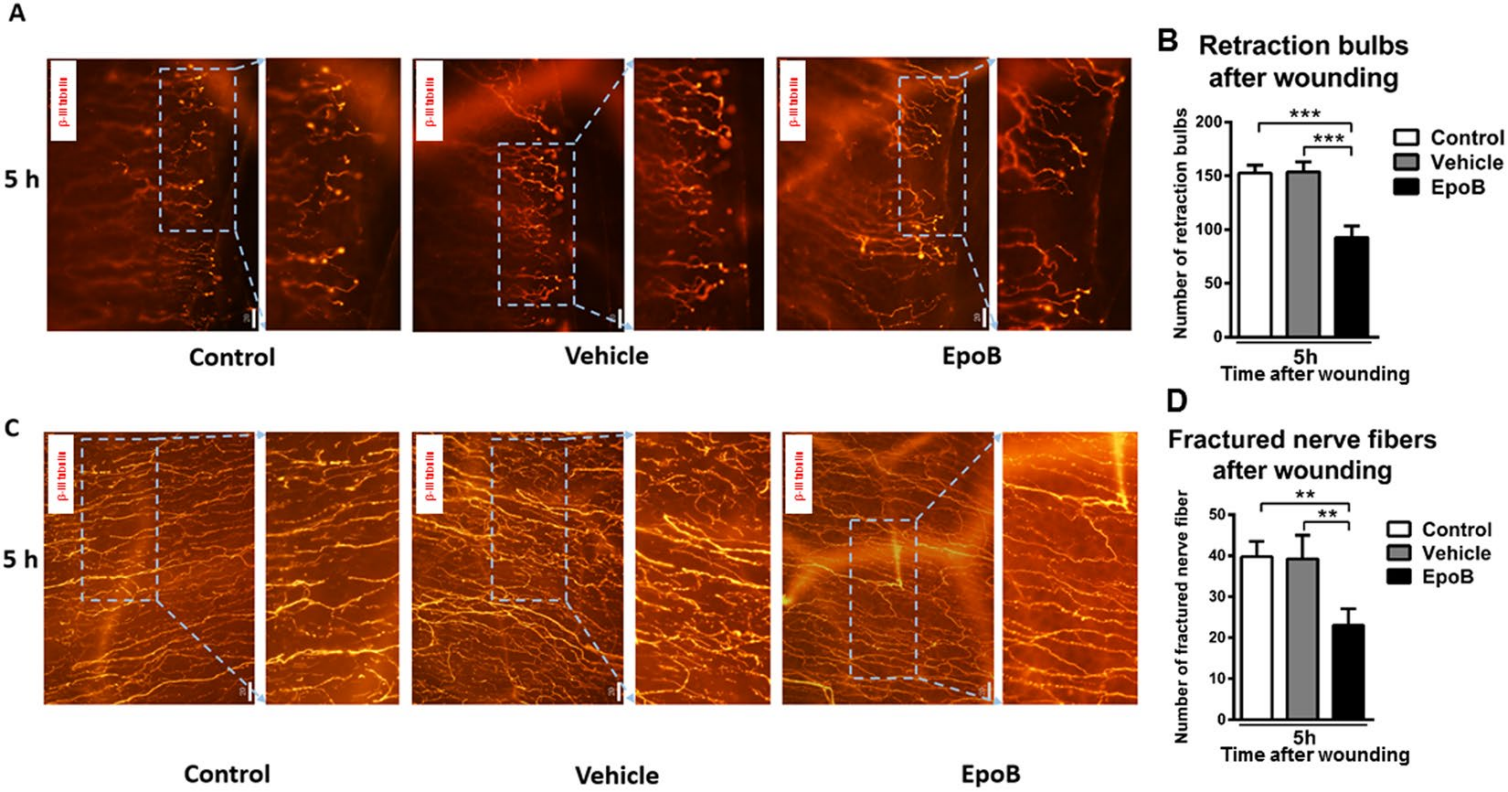
EpoB prevents axons from injury-induced degeneration at an early stage after corneal wounding. **(A)** Representative retraction bulbs in Zone 3, 5 h after abrasion (40X). **(B)** The number of retraction bulbs in Zone 3 in three groups at 5 h after abrasion (40X). **(C)** The nerve fragments in Zone 2 at 5 h after abrasion (40X). **(D)** The number of nerve-fractured fragments in Zone 2 in three groups at 5 h after abrasion (40X). Significant differences were found compared to the vehicle and normal groups (mean ± SD. Student’s *t*-test.) *P < 0.05, **P < 0.01, ***P < 0.001; *n* = 6 corneas/group.

Peripheral nerve injury rapidly initiates a cascade of degenerative cellular and molecular changes at the site of an injury. This active process results in the formation of characteristic swellings at the nerve tips known as retraction bulbs (RBs), as well as the fragmentation and disintegration of the axon^35^. MTs in degenerated axons are dispersed, disorganized, and even lost^21^. To identify any preventive effects of EpoB on injury-induced degeneration, we visualized the dynamic behavior of the subbasal nerve fiber at 5 h after abrasion following the mechanical removal of a 2-mm diameter section of central corneal epithelium. In agreement with a previous publication^17^, evident degeneration was induced rapidly after wounding. Subbasal nerve fibers formed RBs at the wound edge at 5 h following abrasion (Fig. 4A); subsequently, continuous MTs (labeled by a neuron-specific marker, beta-III tubulin) in the injured axons began to gradually break into fragments (Fig. 4C). Interestingly, EpoB pretreatment caused a marked decrease in the number of RBs at the wound edges, from a total of 152.75 ± 7.15 (*n* = 6 corneas) in four Zone 3 s in the control group and 153.75 ± 9.15 (*n* = 6 corneas) in the vehicle group to a total of 92.50 ± 11.12 (*n* = 6 corneas; *P* = 0.0001) in the EpoB group (Fig. 4A and B). Moreover, the number of fragments ranged from a total of 39.75 ± 3.79 (*n* = 6 corneas) in the control group to 39.25 ± 5.78 (*n* = 6 corneas) in the vehicle group and 23.00 ± 4.12 (*n = *6 corneas; *P* = 0.009) in the EpoB group in axons (Fig. 4C and D). These results support the conclusion that EpoB treatment significantly protects axons from injury-induced degeneration.

### EpoB stabilizes the integrity of beta-III tubulin in the cornea after abrasion

**Figure 5:**
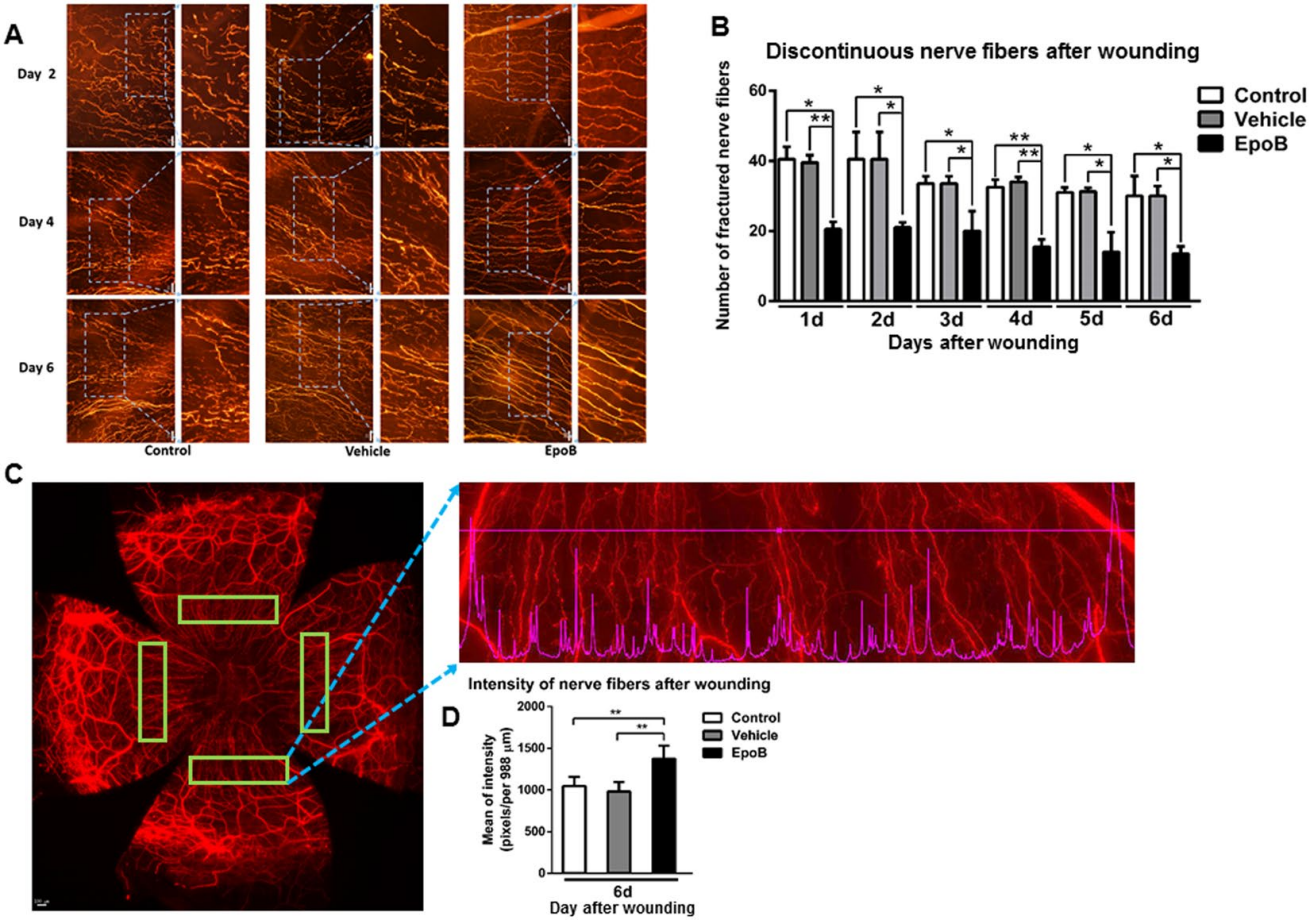
EpoB stabilizes the fiber regrowth in Zone 3 of the cornea after injury. **(A)** Changes in the discontinuous nerve fibers (β-III tubulin staining; original magnification: 400X) in Zone 3 at days 2, 4, and 6 after wounding in the control, vehicle, and EpoB groups. **(B)** The number of discontinuous nerve fibers in Zone 3 from day 1 to day 6 after wounding in the control, vehicle, and EpoB groups. **(C)** and **(D)** Four areas within Zone 3 of the entire whole-mount corneal nerve were selected for the line pixel intensity profile from a row of pixels in the Image window. **(E)** The mean line pixel intensity of nerve fibers at day 6 after wounding in the control, vehicle, and EpoB groups. Significant differences were found compared to the vehicle and normal groups. The results are presented as the mean ± SD. A factorial design ANOVA was performed to analyze the overall differences between the two groups, and Student’s *t*-test was used to compare the differences between the groups by time point. *P < 0.05, **P < 0.01; *n* = 6 corneas/time point.

MTs are integral in guiding axonal growth and repair, and a disorganized MT can impair axon regrowth^36,37^. During the migration of neurons, coordination between MTs and actin filaments in the axon drives the growth cone at the distal end of the axon; however, after axon injury, MTs in the nerve fibers depolymerize and disorganize^21^. Thus, the continuity of the nerve fiber, as shown by a dye-tagged neuron-specific beta-III tubulin antibody, reflects both MT stability and MT dynamics. To assess the effect of EpoB on the stability of beta-III tubulin following an injury, we counted and compared the discontinuous nerve fiber regrowth of the subbasal nerve in Zone 3 at different wounding time points amongst the EpoB pretreated group (treated 1 h before the corneal epithelial abrasion), the vehicle control group, and the normal control group. The data showed that the total number of discontinuous nerve fibers significantly decreased from day 1 to day 6 after corneal abrasion compared to the control and vehicle groups (Fig. 5A and B). To determine whether beta-III tubulin stabilization is enhanced by EpoB, intensity line profiles from the three treatment groups were analyzed using the Intensity Data module of DeltaVision Elite. The results indicated that the nerve fibers in the group treated with EpoB showed significantly higher pixel intensity values (Fig. 5D) compared to the control and vehicle groups. Thus, this suggests that EpoB stabilizes the integrity of beta-III tubulin as well as the state of nerve regrowth in the cornea.

### EpoB increases nerve bead frequency in the cornea after abrasion

**Figure 6:**
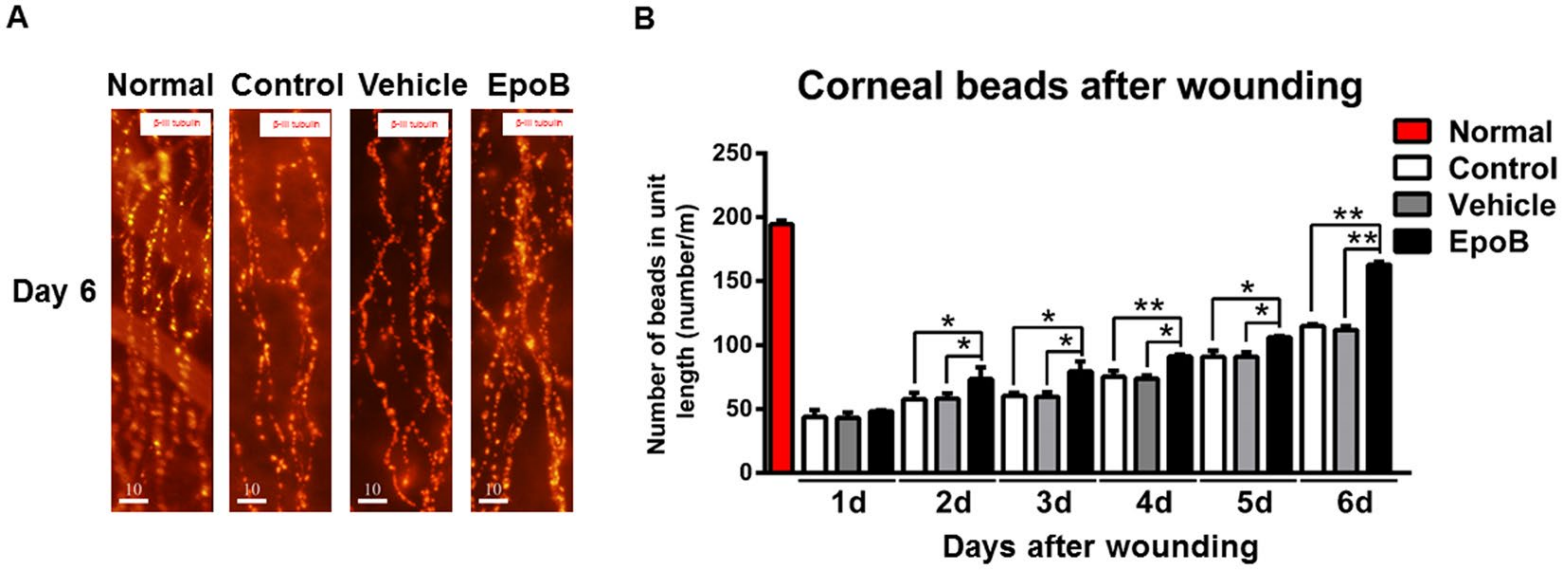
EpoB increases the beading frequency in Zone 3 of the corneal nerve fibers after injury. **(A)** Representative DeltaVision Elite high-resolution microscope images in Zone 3 showing beads in the normal control, vehicle control, and EpoB treatment groups at 6 days postinjury and in the uninjured group (bar = 10 μm, 40X). **(B)** The number of beads in Zone 3 in uninjured corneas and corneas from day 1 to day 6 after corneal abrasion. Significant differences were found compared to the vehicle and normal groups. The results are presented as the mean ± SD. A factorial design ANOVA was performed to analyze the overall differences between the two groups, and a Student’s *t*-test was used to compare the differences between the groups by time point. *P < 0.05, **P < 0.01; *n* = 6 corneas/group.

Corneal nerve fibers are beaded nerve fibers, and within the beads are mitochondria, glycogen particles, and vesicles^38^. Furthermore, the size and number of beads may be associated with the metabolic status of the corneal nerve fibers^39^. Several recent studies have shown that an increase in the number and density of mitochondria in injured axons is critical for axon regeneration due to the generation of adenosine triphosphate (ATP)^40–42^. To roughly evaluate the effect of EpoB administration on the behavior of mitochondria in subbasal nerve fibers after abrasion, we counted and compared the number of beads in Zone 3 amongst the EpoB, vehicle, and normal control groups. While there was no significant difference in the beading frequency amongst the three groups on day 1 or day 2 after corneal abrasion, the beading frequency was significantly increased in the EpoB-treated group from day 3 to day 6 when compared to the normal control and vehicle groups (Fig. 6A and B). These results suggest that EpoB treatment significantly increases the beading frequency of the corneal nerve fibers, which is thought to indicate an increase in metabolism^43^.

### EpoB enhances corneal nerve regrowth and improves the recovery of sensitivity after abrasion

**Figure 7:**
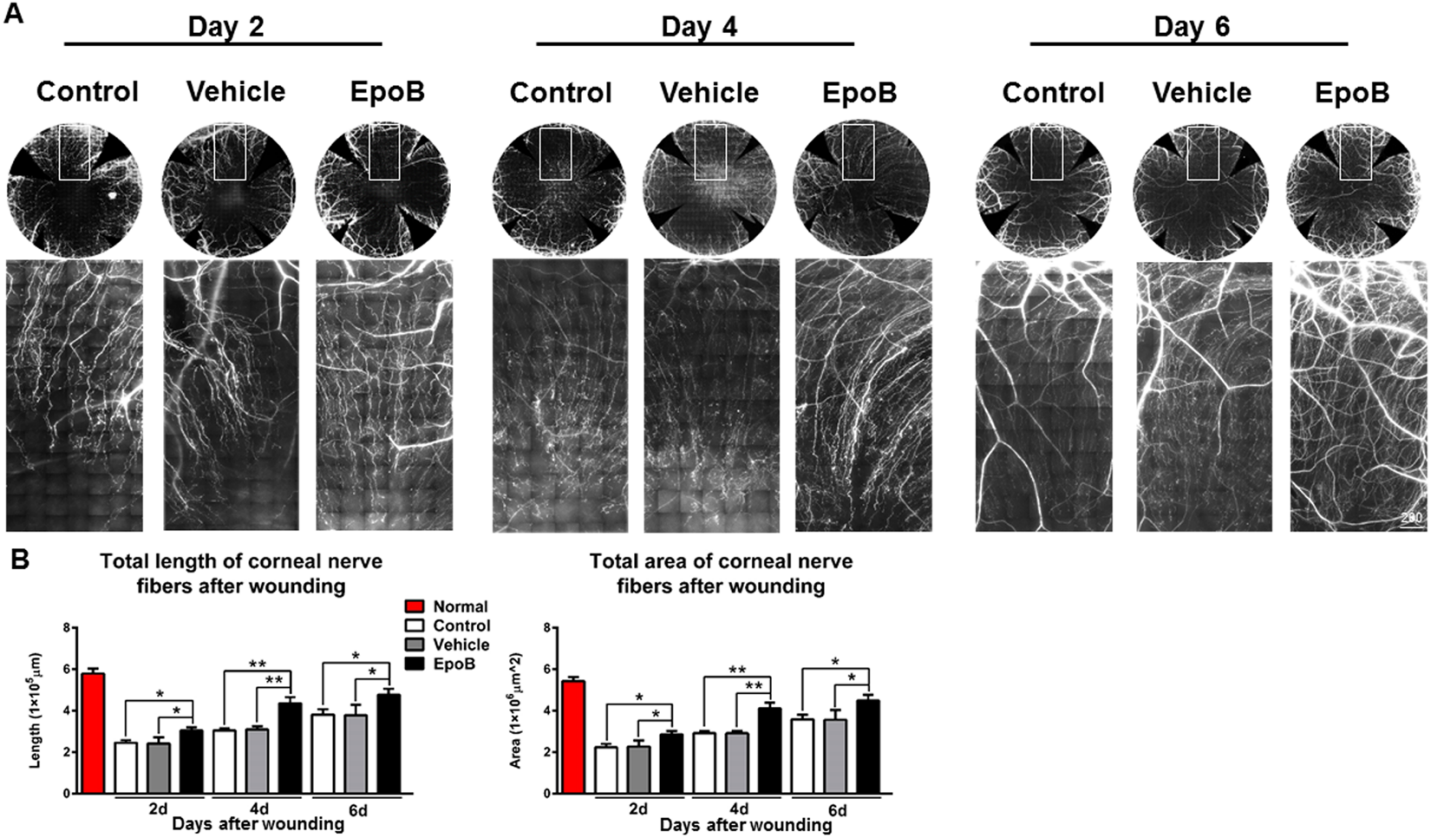
The systemic administration of EpoB promotes corneal nerve regrowth after abrasion. **(A)** Photos of complete corneal nerves scanned using a DeltaVision Elite high-resolution microscope (β-III tubulin staining) and photos of a partially enlarged area. **(B)** Changes in the total length and total area of the nerve fibers of the central test region (Fig. 10B) in the EpoB, control, and vehicle groups. Significant differences were found compared to the vehicle and normal groups. The results are presented as the mean ± SD. A factorial design ANOVA was performed to analyze the overall differences between the two groups, and Student’s *t*-test was used to compare the differences between the groups by time point. *P < 0.05, **P < 0.01; *n* = 6 corneas/group.

Recent evidence indicates that the MT stabilization compounds Taxol and EpoB increase MT dynamics in growth cones and thereby facilitate axon extension in the mouse CNS^21,22,24^. To test whether similar mechanisms could underlie the beneficial effects of EpoB in the injured corneal nerve, we acquired images of nerve fibers in the whole cornea on days 2, 4, and 6 after wounding using a DeltaVision Elite high-resolution microscope (60×) and then measured the total length and total area of the nerve fiber in the central test region (Fig. 10B) with the Imaris 64×image analysis system. Indeed, we found that the total length and total area of the nerve fiber in the central test region (Fig. 10B) in the wounded corneas gradually increased over time in the three groups (Fig. 7A and B). However, the total length and total area of the corneal nerves of the central test region (Fig. 10B) in the group treated with EpoB were significantly higher than those of the control and vehicle control groups on days 2, 4, and 6. This suggests that EpoB is capable of promoting nerve regeneration after corneal abrasion.

**Figure 8:**
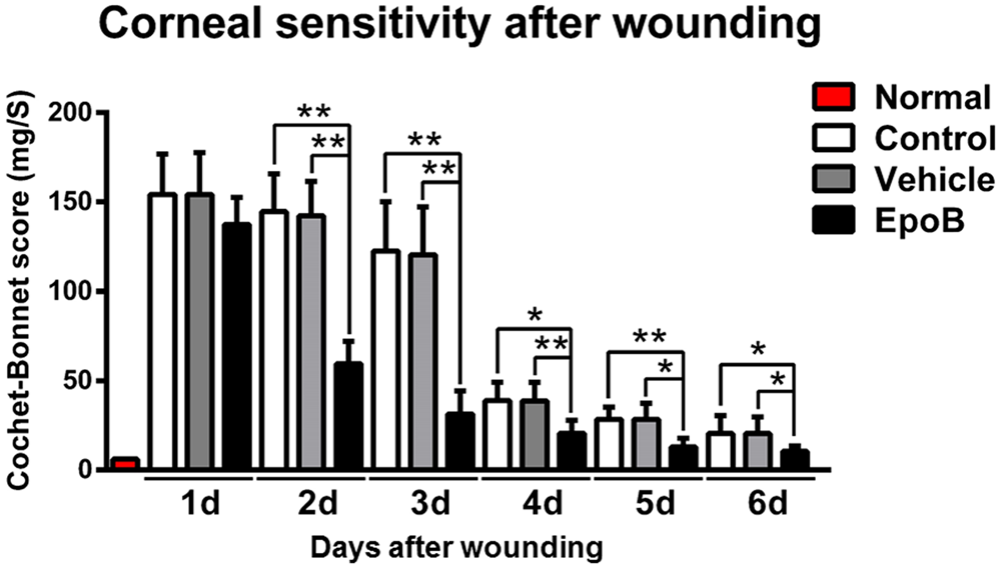
EpoB promotes the recovery of corneal sensitivity. Mouse corneas were scraped with a golf-club-like scraper. At 1, 2, 3, 4, 5, and 6 days after abrasion, the corneal sensitivity levels were measured by a Cochet-Bonnet esthesiometer and compared among the EpoB, vehicle, and normal groups. The results are presented as the mean ± SD. A factorial design ANOVA was performed to analyze the overall differences between the two groups, and Student’s *t*-test was used to compare the differences between the groups by time point. *P < 0.05, **P < 0.01; *n* = 6 corneas/group.

Nerve regeneration is often associated with functional recovery. To determine whether EpoB treatment leads to functional recovery (touch sensitivity) after corneal abrasion, the corneas were monitored for sensitivity from day 1 to day 6 following abrasion. Normal corneas without abrasion exhibited maximum corneal sensitivity with Cochet-Bonnet scale values close to 6 cm (100% sensitivity). At day 1 after abrasion, partial touch sensitivity was recorded, but the difference among the three groups was not significant (Fig. 8); afterwards, the touch sensitivity of the cornea was gradually restored. EpoB treatment markedly increased touch sensitivity on the corneal surface from day 2 to day 6 after abrasion (Fig. 8) compared to the vehicle treatment and normal controls. Thus, EpoB administration promotes functional recovery of the corneal nerve after corneal abrasion, and EpoB-induced functional recovery may correlate with its promotion of axon regrowth.

### Systemic postinjury administration of EpoB improves corneal nerve regrowth and the recovery of sensitivity

**Figure 9:**
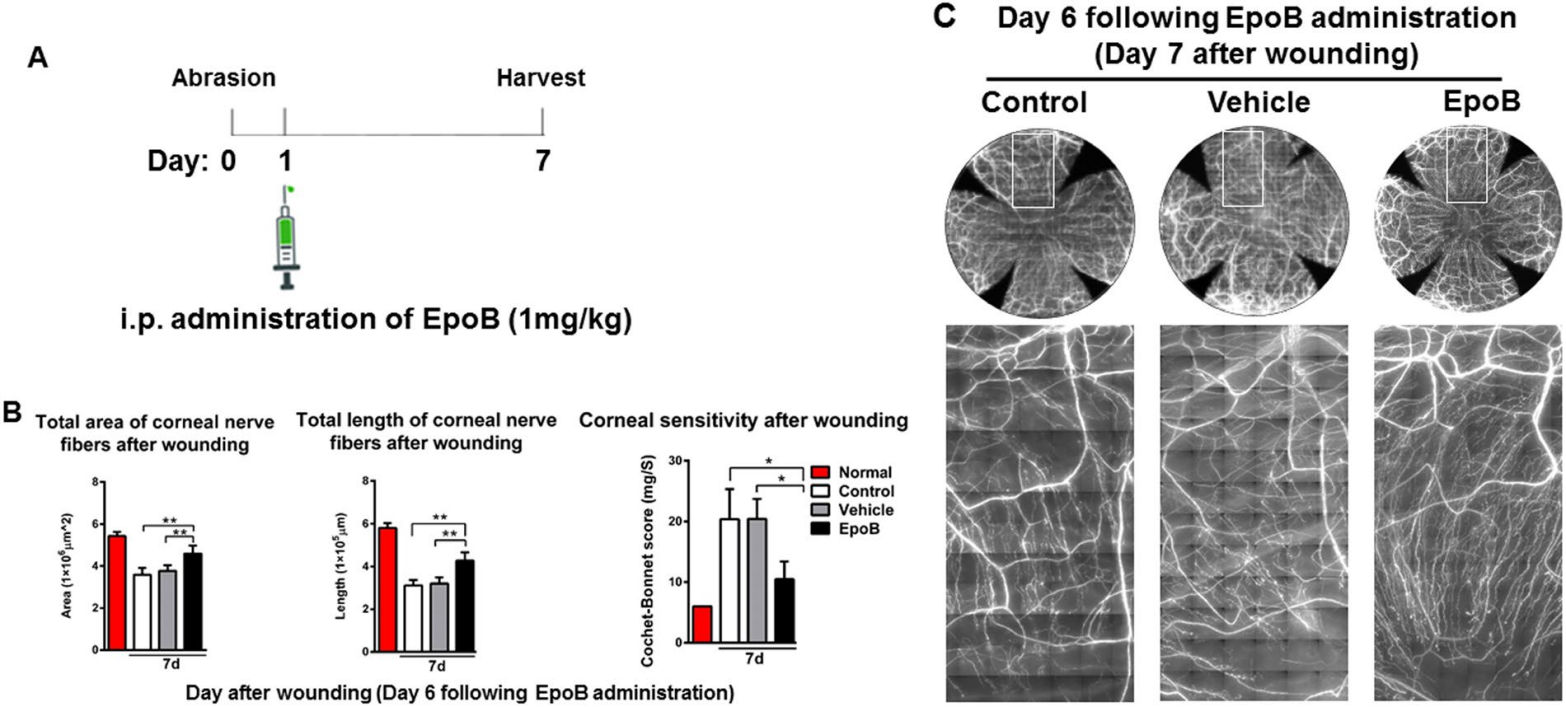
The systemic postwound administration of EpoB improves corneal nerve regrowth and the recovery of sensitivity. Animals were treated with i.p. administration of EpoB 24 hours after corneal epithelium abrasion (**A**). The nerve regrowth area (**B** *left* and **C**), nerve regrowth length (**B** *middle* and **C**), and the sensitivity of the cornea (**B** *right*) at 7 days after corneal abrasion was determined.

The data obtained above showed that pretreatment with EpoB has a significant effect on corneal nerve regeneration and functional repair. To establish the therapeutic effect of EpoB after corneal abrasion, we treated animals with i.p. administration of EpoB (1 mg/kg) at a clinically relevant time point, 24 hours after corneal epithelium abrasion (Fig. 9A), and then assessed the total corneal nerve length and total corneal nerve area of the central test region and the sensitivity. The results showed that both the corneal nerve length (Fig. 9B *left* and C) and area (Fig. 9B *middle* and C) were significantly increased in the EpoB treated group compared to the normal control and vehicle groups. In addition, corneal sensitivity was also significantly improved compared to the control and vehicle groups (Fig. 9B *right*). These results suggest that the early administration of EpoB after corneal abrasion has the potential to improve corneal nerve regeneration and functional recovery.

## Discussion

The rapid recovery of corneal neurological function after infection and injury is critical for visual recovery. However, the intrinsic axon growth capacity of the cornea is limited. Therefore, the search for measures to promote repair is of great importance. Here, we report a marked therapeutic effect of a specific MT stabilizer, EpoB, on reinnervation and functional recovery after injury. We found that the systemic administration of EpoB at an optimized dose attenuates the degeneration of corneal nerve fibers at an early stage after abrasion. More importantly, the systemic administration of EpoB stabilizes corneal nerves and makes them less susceptible to depolymerization or disorganization, which consequently promotes axonal growth to the wound area and notably improves stimulus detection after injury. In addition, we found that the administration of EpoB at 24 hours after corneal abrasion has a significant therapeutic effect on nerve regeneration and functional recovery. Finally, we also demonstrated the rapid distribution and slow elimination of EpoB in both the cornea and trigeminal ganglion after systemic administration. Thus, this study provides an effective potential strategy to promote nerve regeneration in the cornea after wounding.

Previous PK studies of EpoB showed that the drug can be rapidly and widely distributed to large organs, the CNS, and plasma in both mice and rats^29^. In agreement with a previous study^24^, we found that the rate of elimination of EpoB varies based on the organ being studied. In the CNS, cornea, and trigeminal ganglion, the EpoB concentration is relatively constant and is maintained above therapeutic levels for more than 6 days. In contrast, there is a relatively rapid decrease of EpoB in large organs such as the heart, liver, spleen, lungs, and kidneys. We observed that the concentration of EpoB in the cornea and trigeminal ganglion was even higher than in the CNS and was basically constant from day 1 to day 6 after administration. It is assumed that EpoB rapidly spreads to the organs after systemic administration and then returns to the blood. Thus, EpoB may continuously circulate in the ophthalmic artery, and especially in the dilated limbal vessels, which maintain high permeability after corneal injury. Moreover, these drugs deposit in and readily bind to the nerve fibers in the cornea. One study revealed that this is true even when the concentration of EpoB is less than 0.1 ng/ml ^33^. We found that this drug can stay in the cornea and trigeminal ganglion at a concentration of 10 ng/ml for more than 6 days after administration, providing strong evidence for the therapeutic potential of EpoB.

MTs are the building blocks of axons, with axon extension occurring through microtubule assembly. Beyond their role as structural scaffolds, the continuous remodeling of microtubules is vital for axon growth and guidance. *In vivo* imaging of MT dynamics after injury has revealed a rapid dissolution of microtubules at the injury site^44^, which is followed by the upregulation of dynamic MTs and a subsequent increase in MT growth concomitant with growth cone reformation^45^. When MTs are pharmacologically destabilized or disorganized by nocodazole, growth cones retract into bulb-like structures, and axon growth is inhibited^21^. Conversely, the application of the MT-stabilizing drug paclitaxel (Taxol) has been shown to prevent the formation of RBs^21,23^ and promote axon regeneration after nerve injury^22,23^. This suggests that MT stability and dynamics are key determinants for the reconstruction of proper nerve endings and subsequent axon regeneration.

EpoB was recently approved by the FDA as a MT-stabilizing drug for cancer treatment. It can cross the blood-brain barrier and exhibits differential effects on the MT cytoskeleton in neurons and fibroblasts. In neurons, EpoB induces rapid MT polymerization in the neurite tips, promotes axon elongation in the CNS, and shows promise for clinical applications^24^. The corneal nerve in our abrasion model belongs to the peripheral nerve system (PNS). Consistent with the observation in the CNS model, we found that EpoB can promote the early regrowth of subbasal nerve fibers, the extension of nerve fiber endings, and the recovery of sensitivity after corneal abrasion. This suggests that this compound is also effective for peripheral nerve regeneration and functional recovery after injury through the stabilization of MTs.

Recently, several studies have concluded that the increase in mitochondrial density after nerve trauma and the ATP provided by these mitochondria play key roles in axon regeneration^40–42^. Han *et al*.^41^ found that the mitochondrial density doubled after the injury of the axon connecting the ventral and dorsal nerve cords in *C. elegans*. Further studies revealed that 39.2% of damaged axons with high mitochondrial density could regenerate, whereas only 6.6% of axons with low mitochondrial density regenerated. This suggests that increased mitochondrial density in damaged axons is necessary for successful regeneration. Consistent with the high energy requirement for axonal regeneration, worms with impaired mitochondrial respiratory chains and reduced ATP levels showed reduced axonal regeneration. Cartoni *et al*.^42^ found that the mammalian-specific gene armadillo repeat containing X-linked 1 (Armcx1), a critical regulator of mitochondrial transport, was upregulated after axotomy. Moreover, they found that Armcx1 overexpression enhanced adult retinal nerve mitochondrial transport in ganglion cells. Importantly, Armcx1 also promotes neuronal survival and the regeneration of axons after injury, depending on the localization of their mitochondria. Similarly, the energy and regeneration defects in damaged sciatic nerves were restored by the enhancement of mitochondrial transport induced by a loss of mitochondria-anchoring protein syntaphilin^40^. These data indicate that the supply of energy is the main function of mitochondrial transport during axonal regeneration. In our study, we found that a pretraumatic EpoB treatment significantly increased the density of mitochondria in the nerve fibers. Therefore, we hypothesized that the mechanism by which EpoB accelerates corneal nerve growth may be related to the stabilization of MTs, which facilitates accelerated mitochondrial migration into the traumatized nerve and thereby provides the energy for repair. However, the detailed mechanisms underlying these effects need to be further studied and confirmed.

Despite EpoB’s effects on the improvement of reinnervation and restoration of sensitivity, it should be noted that epothilones have some potential to induce peripheral neuropathy by interfering with axonal transport and cytoplasmic flow in affected neurons and through the disruption of MTs of the mitotic spindle^46–48^. Therefore, there is clearly a need for further and more specific experimental studies to screen for neurotoxicity in epothilone-treated animals. It should be noted that in terms of the dosage and the corneal wound model used in the present study, there seemed to be no significant neurotoxicity or interference with the corneal wound healing process. This conclusion is consistent with the observation in the spinal injury model that there were no systemic changes such as body weight or number of white blood cells, nor were there local changes such as glial fibrillary acidic protein expression, lesions, cell proliferation, or apoptosis at the lesion site^24^.

Accumulating evidence suggests that the administration of MT stabilizers can promote axon regeneration and improve functional activity^21,22,24,45^. Our results demonstrate that the systemic administration of EpoB promotes the regrowth of corneal nerves without obvious adverse side effects in mice following corneal abrasion. Furthermore, EpoB not only accelerates the regrowth of corneal nerves by stabilizing MTs after corneal epithelial abrasion, but also promotes their functional recovery. In addition, the PK study shows that EpoB is stable in the cornea and trigeminal ganglion for a long period. Therefore, EpoB holds potential clinical value for the promotion of nerve fiber regrowth after wounding. However, this study observed only the effects of the systemic administration of EpoB. To minimize unwanted side effects while maximizing efficiency, the next step is to develop an effective EpoB formulation for topical absorption. In addition, this study also certified the therapeutic effects of the administration of this drug within 24 hours after wounding. Altogether, our data suggest that there is therapeutic potential of EpoB for corneal nerve regrowth and functional recovery after an injury.

## Methods and Materials

### Animals

C57/BL6 mice (8–9 weeks old) free from eye diseases were purchased from the Animal Experimental Center of Guangdong Province. The body weight of the mice ranged from 18 to 20 g. All the animal protocols were approved by the Jinan University Laboratory Animal Committee for Animal Welfare, and the animals were treated in accordance with the guidelines established by the Use of Animals in Ophthalmology and Vision Research Committee and by the Animal Experimental Committee at Jinan University. In this study, only the right eye was injured, and the left eye of each animal served as the control eye.

### Drug Dose

EpoB was purchased from Selleck Chemicals (Shanghai, China), dissolved in DMSO at a concentration of 20 mg/ml, and stored at −20 °C. The mice were randomly divided into 3 experimental groups (6 corneas analyzed per group): the normal control group, the intraperitoneal (i.p.) administration of 1 mg/kg EpoB group with no obvious adverse side effects^24^, and the mock treatment group (injected with the DMSO vehicle only). The animals were pretreated with EpoB and/or the vehicle 1 h before injury.

### Pharmacokinetic Analysis

EpoB was dissolved in a cosolvent composed of DMSO:saline (1:200 V/V). C57/BL6 mice (*n* = 5) were given EpoB by i.p. injection at a dosage of 1 mg/kg. At predetermined time points (6, 12, 24, 36, 48, 60, 72, 84, 96, 108, 120, 132, and 144 h), the animals were anesthetized by an i.p. injection of 10% chloral hydrate (0.05–0.1 ml), and the blood was removed by a heart puncture followed by a whole-body flush with 0.9% saline containing 20 U heparin sodium. The whole blood was centrifuged at 9,000 *g* for 10 min to separate the plasma. All the major organs (brain, spinal cord, heart, liver, spleen, lungs, and kidneys), the cornea^17,49^, and the trigeminal ganglion^50^ were also collected and stored at −80 °C until analysis.

EpoB was quantified by liquid chromatography–mass spectrometry (LC-MS; UPLC-QTOF/MS system, Waters, Milford, MA) in accordance with a previous publication^51^. Epothilone A was used as the internal standard (IS). Standard stock solutions of EpoB and the IS were prepared in DMSO at 2.0 mg/ml and stored at −20 °C. The EpoB stock solution was diluted using DMSO/methanol (1/1 v/v) in five serial working solutions with concentrations ranging from 302.5 to 5000 ng/mL. The tissue samples were weighed, and a volume of saline twice the volume of the tissue was added. Then, the mixture was homogenized.

The calibration curve was prepared as follows: 10 μL of EpoB working solution was added to 90 μL blank mouse plasma or tissue homogenate to obtain a standard curve, with the final concentrations ranging from 30.25 ng/ml to 500 ng/mlL. Then, 500 μL of IS working solution (1 μg/ml) was added to the mixture, which was then vortexed for 10 s. The mixture was centrifuged at 9,000 *g* for 10 minutes, and the supernatant was transferred to a clean tube and evaporated to dryness under a stream of nitrogen using an evaporator. Finally, the dried extract was reconstituted in 100 μL of water/acetonitrile (50/50 v/v diluent). As with the standard curve preparation, 100 μL of plasma or tissue homogenate was transferred to a 1.5-ml test tube, and the same steps were followed.

### Corneal Epithelial Abrasion Model

Central corneal wounding was performed by following our previously described method^49^. Briefly, the mice were anesthetized by an i.p. injection of 10% chloral hydrate (0.05–0.1 ml). Under a stereomicroscope, the central corneal epithelium was marked with a 2-mm-diameter trephine, and then the marked epithelial cell layer was mechanically scraped using a golf-club-like spud (Accutome, Malvern, PA). Artificial tears were dropped onto the ocular surface to prevent dryness around the wound area. Wound closure was assessed using fluorescein staining of the ocular surface. The wounded corneas were photographed under a dissecting microscope at postwound hours 0, 6, 12, 18, and 24 using a fixed camera lens. The measurements and digital analyses of the wound areas were performed using Adobe Photoshop CS2 software to compare the pixels in the histogram function.

### Immunofluorescent Staining

The corneal nerves were stained using a standard method described elsewhere^2^. Briefly, the eyeballs were removed under a dissecting microscope and fixed in 2% paraformaldehyde in phosphate-buffered saline (PBS) for 1 h. The corneas, along with the complete limbus tissues, were trimmed from the eyeball in PBS, blocked in 0.1 M PBS containing 2% ACN (BSA) for 15 minutes, and permeabilized with 1% Triton X-100 in BSA/PBS for 15 minutes. Then, the corneas were incubated in 0.1 M BSA/PBS and 0.1% Triton X-100 with neuron-specific beta-III tubulin NorthernLights NL557-conjugated antibody (Cat# NL1195R, R & D Systems Inc., Minneapolis, MN, USA) and FITC-conjugated anti-Ly6g (Cat#:11–9668–82; Clone:1A8-Ly6g, 3:100; eBiosciences, USA) for 24 h at 4 °C so that the corneal nerve fibers and neutrophils, respectively, could be observed. After incubation, the tissues were washed in 0.1 M PBS 3 times for 5 min each time. Finally, the corneal buttons were cut radially into four quadrants, stretched, mounted using an antifade mounting medium containing 4’,6-diamidino-2-phenylindole (DAPI) (Sigma-Aldrich, USA), and stored in the dark at 4 °C.

### Computational Analysis of Corneal Nerve Fibers

**Figure 10:**
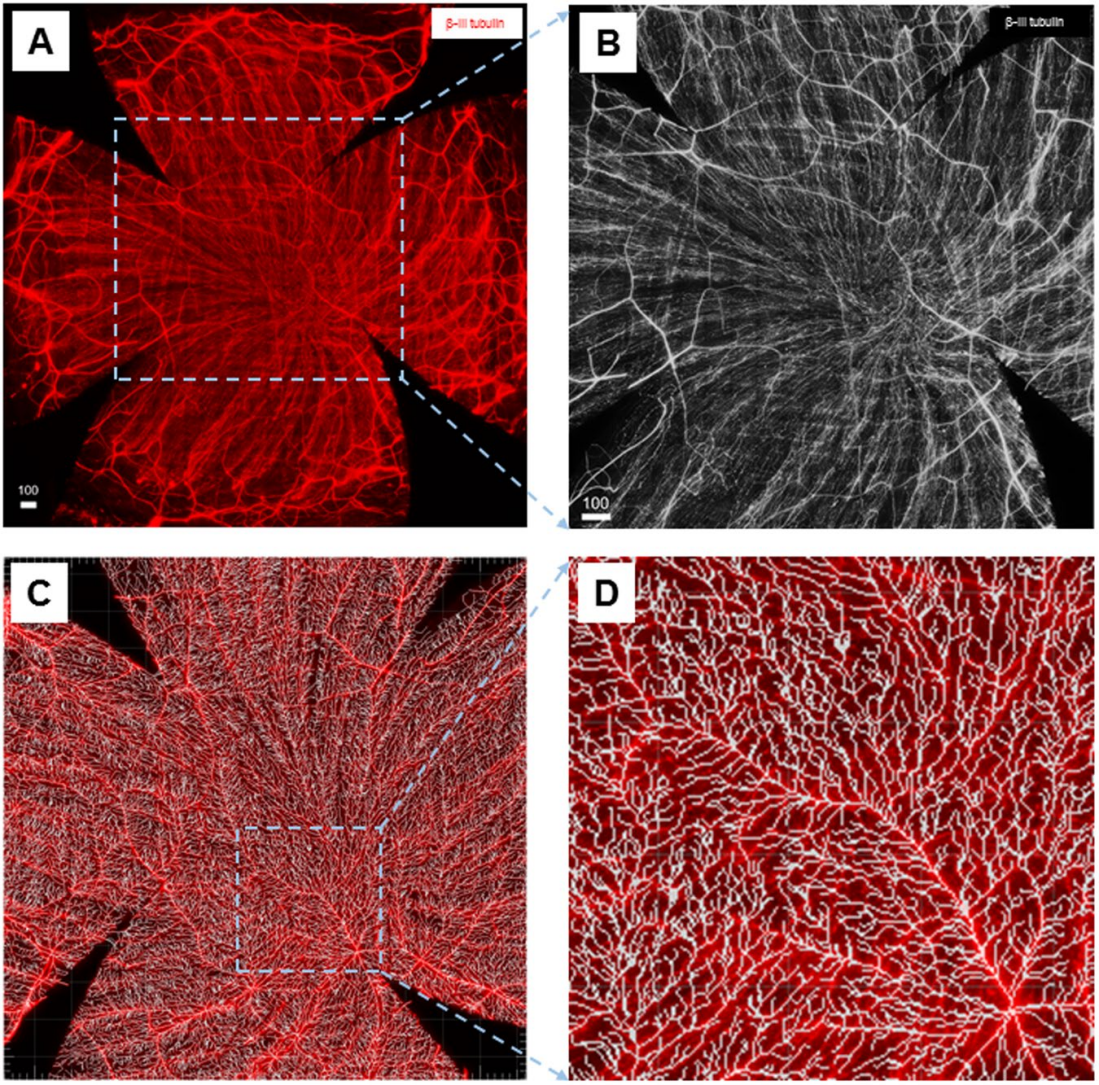
The central test region of the whole-mount view of the subbasal nerves for the analysis of normal corneal nerves and regrown nerves after abrasion using the Imaris system. **(A)** Whole-mount view of corneal nerves after beta-III tubulin staining showing all the corneal nerve fibers. **(B)** 2000 μm × 2000 μm central test region located in the central area of the cornea for the nerve regrowth analysis. **(C)** The image after the Filament Tracer module treatment. **(D)** Further magnification of the highlighted smaller area from the 2000 μm × 2000 µm analysis area.

Images of whole-mounted corneal nerve fibers were obtained by stitching together individual stack images acquired using a DeltaVision Elite high-resolution microscope image system under a 60X objective. To avoid interference with the vertical nerve fibers in the epithelium, the subbasal fiber dimension was focused on first at the start of the z-dimension scanning under 60X magnification. All the image files were deconvolved in the deconvolution module to obtain high-quality image signals. The stitched images were further processed using Imaris 6.2 software (Bitplane AG, Zurich, Switzerland) to calculate the fiber length and area. First, a 2000 µm × 2000 μm area in the central test region (Fig. 10B) was selected from the entire corneal nerve image (Fig. 10A) for the analysis of nerve fiber regrowth after abrasion. Then, the Filament Tracer module was used for the detection and visualization of nerve fibers (Fig. 10C and D). The total length and total area of the nerve filaments of the central test region (Fig. 10B) were calculated and obtained by adjusting the Dendrite and Seed Points Threshold modules. For some stitched whole-mount corneal nerve images, four sites (988 µm × 251 μm; Figs. 5C, 4 yellow squares) in Zone 3 region of each of four quadrants were selected. The intensity line profiles, which are plots of intensity values obtained from a row of pixels in the Image window, were analyzed using the Examining Intensity Data module of DeltaVision Elite to compare the level of beta-III tubulin present in the images (Fig. 5D). These data were exported from the Data Inspector as a text file, and the statistics were calculated using the Data Inspector Statistics.

### Measurement of Corneal Sensitivity

To analyze the functional recovery of corneal sensation, corneal sensitivity after wounding was measured using a Cochet-Bonnet esthesiometer (catalog no. 8630-1490-29; Luneau SAS, France), as published previously^52^. Briefly, at different times after abrasion, unanesthetized mice were held by the scruff of the neck and presented with a monofilament at lengths ranging from 6.0 to 0.5 cm in 0.5-cm increments to elicit a blink response. At each length, the monofilament touched the cornea four times, making perpendicular contact with the surface before considering a response to be negative (no blink response). The lack of a blink reflex at a monofilament length of 0.5 cm was recorded as “0.” All the measurements were performed by the same examiner, who was blinded to the animal groups.

### Counting and Analysis of Neutrophils, Epithelial Division, Fractured Nerve Fragments, and Beads

**Figure 11:**
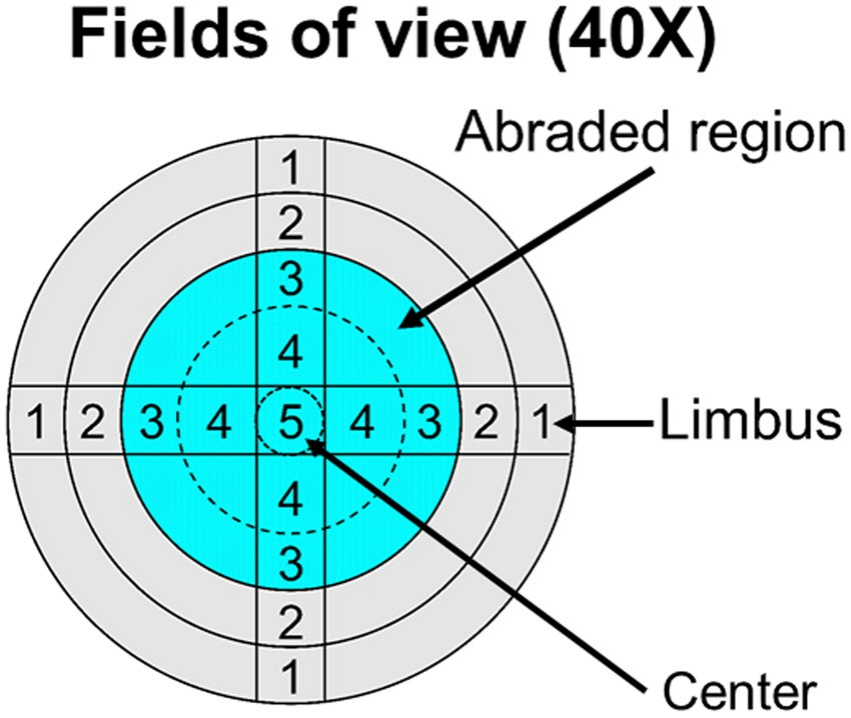
Diagram showing the microscopic zones examined during the analysis of the cornea. The analysis was performed by counting specific parameters: degenerated and fractured fibers from Zone 2 and beads of the corneal nerve wound edge from Zone 3 at 5 h after wounding; fractured fibers of corneal fiber regrowth from Zone 3 from day 1 to day 6 after wounding; Ly6g+ neutrophils in Zone 4 at different time points after corneal abrasion; and dividing cells from limbus to limbus (total of 9 zones) at different time points after corneal abrasion, shown in the figure at 40×, as indicated in each of the four regions of the cornea. The blue area represents the original central wound area with epithelial removal.

As shown in Fig. 11, the cornea was divided into five zones of view: Zone 1, Zone 2, Zone 3, Zone 4, and Zone 5 from limbus to center. A zone refers to an entire field of view at 40 × magnification, for which we acquired three-dimensional images of size 1024 µm × 1024 µm. To compare the difference in the abundance of neutrophils (Ly6g+ cells) infiltrating the wound area among the different groups, a total of four Zone 4 s, as shown in Fig. 11, were collected as representative wound areas with neutrophil infiltration. The neutrophil number in the whole corneal thickness was manually counted step-wise by adjusting the scroll arrow of the dialog box in the opened file. An assessment of cell division in the corneal epithelium was performed in corneal whole mounts and determined by staining with FITC-conjugated monoclonal anti-α-tubulin (1:200, clone DM1A, Sigma-Aldrich) to label spindle MTs during mitosis and with rhodamine-labeled phalloidin (1:50, Cat# R415, Invitrogen) to label F-actin, as described previously^53^ (Fig. 5E). To compare the degeneration of nerve fibers at the early stage of wounding after abrasion among different groups, subbasal nerve fiber images from Zone 2 were taken, and the number of discontinuous nerve fragments was manually counted.

Lesioned axons form characteristic swellings at their tips known as retraction bulbs, which are caused by the destruction of MTs, suggesting that growth is inhibited^21^. To compare the effects of EpoB on retraction bulb formation, the number of retraction bulbs at Zone 3 (the leading edge of the wound) was counted. To compare the effects of EpoB on the nerve bead number, the beads in the nerve at Zone 3 (the stable nerve growth area) on days 1, 2, 3, 4, 5, and 6 post-corneal abrasion were enumerated under the microscope (40X). The nerve bead frequency was represented by the number of beads per unit length (in meters) of the nerve fibers in the measured area. To compare the stability of alpha-III tubulin visualization at days 1, 2, 3, 4, 5, and 6 postabrasion, subbasal nerve fiber images at Zone 3 were taken under the microscope (40X), and the number of discontinuous nerve fragments was manually counted. All the counting work was performed by the same person.

### Statistical Analyses

The data were analyzed using SPSS 21.0 software and are presented as the means ± the standard deviation. A factorial design analysis of variance (ANOVA) was used to compare the overall differences between groups, and a Student’s *t*-test was used to analyze the differences between groups at each time point. A *P* value < 0.05 was considered significant. The PK parameters were obtained using WinNonlin 3.0 Professional.
